# Rehabilitation Profiles of Older Adult Stroke Survivors Admitted to Intermediate Care Units: A Multi-Centre Study

**DOI:** 10.1371/journal.pone.0166304

**Published:** 2016-11-09

**Authors:** Laura M. Pérez, Marco Inzitari, Terence J. Quinn, Joan Montaner, Ricard Gavaldà, Esther Duarte, Laura Coll-Planas, Mercè Cerdà, Sebastià Santaeugenia, Conxita Closa, Miquel Gallofré

**Affiliations:** 1 Convalescence and Rehabilitation Unit, Hospital Parc Sanitari Pere Virgili, Barcelona, Spain; 2 Department of Medicine, Universitat Autónoma de Barcelona, Barcelona, Spain; 3 Institute of Cardiovascular and Medical Sciences, University of Glasgow, Glasgow, United Kingdom; 4 Neurology Department, Neurovascular Research Laboratory (VHIR), Vall D´Hebrón Hospital, Barcelona, Spain; 5 Department of Computer Science, Universitat Politécnica de Catalunya, Barcelona, Spain; 6 Physical Medicine and Rehabilitation Department, Parc de Salut Mar, Barcelona, Spain; 7 Fundació Salut i Envillement, Universitàt Autónoma de Barcelona, Barcelona, Spain; 8 Catalan Healthcare Service, Government of Catalonia, Barcelona, Spain; 9 Department of Geriatric Medicine and Palliative Care, Badalona Serveis Assistencials, Badalona, Spain; 10 Rehabilitation Department, Corporación Fisiogestión, Barcelona, Spain; 11 Pla Director Malaltia Vascular Cerebral, Department of Health, Government of Catalonia, Barcelona, Spain; Yokohama City University, JAPAN

## Abstract

**Background:**

Stroke is a major cause of disability in older adults, but the evidence around post-acute treatment is limited and heterogeneous. We aimed to identify profiles of older adult stroke survivors admitted to intermediate care geriatric rehabilitation units.

**Methods:**

We performed a cohort study, enrolling stroke survivors aged 65 years or older, admitted to 9 intermediate care units in Catalonia-Spain. To identify potential profiles, we included age, caregiver presence, comorbidity, pre-stroke and post-stroke disability, cognitive impairment and stroke severity in a cluster analysis. We also proposed a practical decision tree for patient’s classification in clinical practice. We analyzed differences between profiles in functional improvement (Barthel index), relative functional gain (Montebello index), length of hospital stay (LOS), rehabilitation efficiency (functional improvement by LOS), and new institutionalization using multivariable regression models (for continuous and dichotomous outcomes).

**Results:**

Among 384 patients (79.1±7.9 years, 50.8% women), we identified 3 complexity profiles: a) Lower Complexity with Caregiver (LCC), b) Moderate Complexity without Caregiver (MCN), and c) Higher Complexity with Caregiver (HCC). The decision tree showed high agreement with cluster analysis (96.6%). Using either linear (continuous outcomes) or logistic regression, both LCC and MCN, compared to HCC, showed statistically significant higher chances of functional improvement (OR = 4.68, 95%CI = 2.54–8.63 and OR = 3.0, 95%CI = 1.52–5.87, respectively, for Barthel index improvement ≥20), relative functional gain (OR = 4.41, 95%CI = 1.81–10.75 and OR = 3.45, 95%CI = 1.31–9.04, respectively, for top Vs lower tertiles), and rehabilitation efficiency (OR = 7.88, 95%CI = 3.65–17.03 and OR = 3.87, 95%CI = 1.69–8.89, respectively, for top Vs lower tertiles). In relation to LOS, MCN cluster had lower chance of shorter LOS than LCC (OR = 0.41, 95%CI = 0.23–0.75) and HCC (OR = 0.37, 95%CI = 0.19–0.73), for LOS lower Vs higher tertiles.

**Conclusion:**

Our data suggest that post-stroke rehabilitation profiles could be identified using routine assessment tools and showed differential recovery. If confirmed, these findings might help to develop tailored interventions to optimize recovery of older stroke patients.

## Introduction

Almost 75% of strokes occur in people over 65 years old, with a consequent very high prevalence of older adult stroke survivors with subsequent disability and dependence [1]. This group often has comorbidity and pre-stroke reduced functional capacity, which increases the risk of disability, institutionalization and death [2]. Structured clinical pathways can aid the delivery of evidence-based effective stroke care at all stages of stroke recovery. During the post-acute phase, patients require comprehensive and multidisciplinary care to achieve the best possible functional outcomes. Various models for providing this care have been described, with no consensus on the optimal approach [3].

In the Spanish region of Catalonia, after an acute stroke, patients can be discharged to an in-hospital Intensive Rehabilitation Program (IRP), to an Intermediate Care (IC) unit, to a long-term care facility or to home with community rehabilitation support [4]. IRPs were developed for a specific patient group: low comorbidity, previously independent in activities of daily living (ADL) and with good functional prognosis. IC units serve a less defined patients’ group, generally older, excluded from IRPs and unable to return home directly from the acute hospital for different reasons (comorbidity, medical complications, disability, lack of social support, etc.) [4,5], therefore providing higher proportion of post-acute care than IRP.

Stroke recovery is heterogeneous and is associated with a wide range of factors (including age, stroke severity, comorbidity, disability, access to acute treatment, cognitive function) [6–8]. This fact, together with the limited evidence around post-acute treatment [9,10], complicates the creation of standardized practice and guidelines [11]. A better understanding the case-mix and outcomes for older adults in post-acute stroke rehabilitation could help clinicians to allocate effective interventions, to guide patients and families in setting realistic goals and to assist policy makers in determining resources allocation.

We aimed to identify possible rehabilitation profiles of older adult stroke survivors, based on routine demographic, clinical and social characteristics at admission to IC units, and to describe their outcomes at discharge, also testing differences across profiles.

## Methods

### Design and population

From January to December of 2010, we conducted a multicenter cohort study designed to describe patients’ characteristics and resources utilization in the IC units of Catalonia, Spain. The study was promoted by the Socio-Sanitary and the Cerebrovascular Diseases Master Plans of the Health Department of Catalonia, and was approved by the Animal and Human Experimentation Ethics Committee of the Universitat Autónoma de Barcelona. All patients and/or their family who meet inclusion/exclusion criteria received an explanation about the aims and implication of the study. Written informed consent was obtained from each patient and/or their family. This study was performed in accordance with the Declaration of Helsinki.

Our sampling frame was based on local population size and stroke incidence: each of the 5 peripheral Health Regions of Catalonia was represented by the largest IC unit of the area. For the Health Region of Barcelona, the largest, 5 units were select. We included patients 65 years old and over, admitted to IC during 2010 from any acute hospital, with stroke as primary diagnosis. We excluded patients under 65 years old and those who declined to give informed consent.

### Baseline evaluation

An experienced and trained nurse or physiotherapist, according to staff availability at each site, collected demographic (age and sex), clinical and functional characteristics, as well as aspects describing the healthcare process. Medical information was collected from electronic records and confirmed by the staff. Clinical assessment included: comorbidity (smoking and alcohol consumption, dementia, cerebrovascular disease, diabetes mellitus and dyslipidemia), clinical characteristics at IC admission (pressure ulcers, nasogastric feeding tube, percutaneous enteral gastrostomy, dysphagia and aphasia), the Charlson index [12] and stroke characteristics (type [ischemic/hemorrhagic] and severity at IC admission [National Institute of Health Stroke Scale (NIHSS)] [13,14]. Function was assessed using the Barthel index (BI, score 0–100, disability-independence) [15]. Pre-stroke function was report by patient/caregiver, and post-stroke functional assessment was based on staff observation at IC admission and discharge. We assessed cognitive function using the “Rancho Los Amigos Scale” (RLAS), which evaluates consciousness and cognitive level (score from 1–8 points, coma-intact cognition) [16]. We collected information on pre-stoke residence and presence of a caregiver. Healthcare process variables included beginning rehabilitation at the acute hospital and IC rehabilitation intensity (hours/day and days/week), length of stay (LOS) in IC and discharge destination.

### Outcomes

#### Functional outcomes

(a) Absolute functional improvement (BI at IC discharge minus BI at IC admission) [17]. In addition to the continuous variables, we also considered an improvement of ≥ 20 points in the BI as clinically relevant [18]; (b) Relative functional gain, Heineman or Montebello index (Absolute functional improvement divided by (Pre-stroke BI minus BI at admission)), which calculates the relative functional gain, normalized for the amount of lost function due to stroke as a maximum possible gain [17,19]. It was expressed as a continuous variable and also dichotomized into the best Vs other two tertiles (Heineman 0,6 points or higher Vs lower).

#### Efficiency outcomes

(a) LOS (days). Besides being expressed as a continuous variable, it was also dichotomized as the lowest Vs other two tertiles (cut-point set at 36 days or lower Vs higher; (b) Rehabilitation efficiency (Absolute functional improvement divided by LOS). It was also stated as a dichotomous variable, using best Vs other two tertiles (0.48 points or higher Vs lower); (c) New institutionalization at discharge from the IC unit.

#### Statistical analysis

We described baseline characteristics of the sample, presented as mean values ± standard deviation (SD) for continuous variables, median values ± interquartile range (IQR) for ordinal variables and numbers (percentages) for dichotomous variables.

To identify possible rehabilitation profiles, we performed a k-means cluster analysis using the free access WEKA software, created by the University of Waikato-New Zealand [20]. Cluster analysis has been used in other populations to describe homogeneous sub-groups (clusters) with similar characteristics between them (intra-cluster distance minimized), but different from other groups (inter-cluster distance maximized) [21,22]. The k-means cluster analysis is a partition cluster approach, which divides the sample into smaller non-overlapping sub-groups based on given parameters. Each sub-group must be associated with a “center sub-group point” (based in mean and mode for continuous and categorical variables respectively), and each patient is assigned to the closest center. We included, as “given parameters”, variables previously reported as predictors of functional outcomes or being clinically relevant: age, functional status before and after stroke (BI), Charlson Index, stroke severity (NIHSS), cognitive status (RLAS) and caregiver presence [8,23–27]. The k-means analysis requires to set, a priori, the number of k clusters to be formed [28]. We explored possible clusters solutions by a repetitive analysis for 3, 4, 5, 6 and 7 clusters. After assessing the results of each analysis, we selected the 3 clusters model because larger numbers did not significantly improve the predictive power of the cluster model.

With the aim to give a more operational and informative view of the profiles found through the cluster analysis, and in order to propose a practical tool for clinicians to ideally assign a recently admitted patient to one complexity rehabilitation profile, we complemented the analysis using a decision tree approach using the C4.5 method. We selected C4.5 because, in contrast with other decision tree methods, it allows for categorical variables in the leaves, which in our case is the cluster or complexity profile assigned to a patient. To ensure the validity of the decision tree, we explored the agreement between the complexity profile of each subject using the cluster analysis and the decision tree.

We compared the resulting patient clusters for baseline characteristics not included in the cluster analysis. We also compared the compare the results of each cluster in relation to our functional and efficiency outcomes, using first ANOVA (and ANCOVA, adjusted by sex) and chi-square for continuous and categorical outcomes respectively, in order to obtain a first estimate of the distribution of the outcomes across groups. In both cases, pair-wise comparisons between clusters were performed based on the Bonferroni method. We also performed multivariable regression models to test the association between the clusters and the different selected outcomes. The analysis was adjusted for variables considered to have a potential influence on the outcomes, but not used to create the clusters (stroke type, beginning rehabilitation on acute hospital, presence of dysphagia, and sex). Linear regression was used for continuous outcomes (functional improvement, Heineman, LOS and rehabilitation efficiency) and logistic regression for new institutionalization. We also used logistic regression to test the association between the clusters and dichotomized functional and efficiency outcomes, in order to express the magnitude of the association in a clearer quantitative fashion, using Odds Ratios. To obtain dichotomous variables from continuous outcomes, we used a validated cut-off (functional improvement) when available, and, in other cases, the best vs lowest tertiles (Heineman, rehabilitation efficiency and LOS). Specific cut-offs were mentioned in the outcomes paragraph.

Statistical analyses were performed using SPSS version 19.0 software (IBM Corporation).

## Results

**Fig 1:**
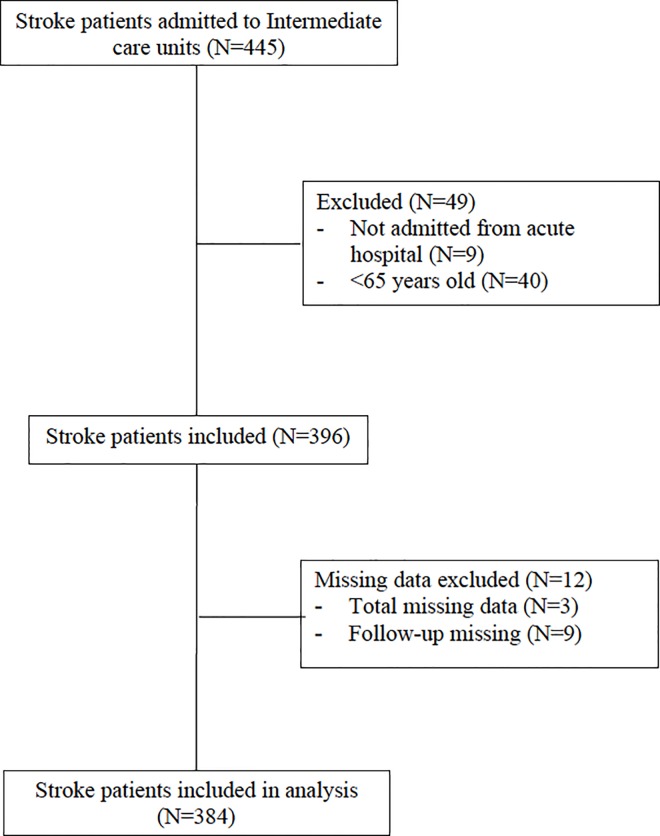
Inclusion and Exclusion chart.

Out of the 10 IC units invited to participate, one withdrew, resulting in 9 IC included. We assessed 445 post-stroke patients; of these, 61 (13.7%) were subsequently excluded because they did not meet inclusion criteria or had relevant missing data (Fig 1). The 384 included participants (mean age±SD 79.1±7.9 years, 50.8% women), had a good pre-stroke functional status (median pre-stroke BI = 100, IQR = 80–100) and moderate comorbidity (median Charlson index = 3, IQR = 1–4), but showed relevant post-stroke disability (median BI at admission = 20, IQR = 5–45), stroke severity (median NIHSS = 9, IQR = 4–15) and cognitive impairment (median RLAS = 7, IQR = 5–8) at admission. The mean±SD LOS in IC was 61.6±45.6 days and 148 patients (48%) had started rehabilitation in the acute hospital Table 1.

**Table 1:** Sample description.

Variables	Total sample N = 384
Age (years)	79.6±7.9
Female	195 (50.8%)
Caregiver present	283 (73.3%)
Smoke consumption	148 (38.5%)
Alcohol consumption	52 (133.5%)
Dementia	79 (20.6%)
Cerebral-vascular disease	129 (33.6%)
Diabetes Mellitus	141 (36.7%)
Dyslipidemia	169 (44.0%)
Previous institutionalization	11 (2.9%)
Charlson Index	3 (1–4)
Ischemic stroke	311 (81%)
Stroke severity (NIHSS)	9 (4–15)
Pre-stroke Barthel Index	100 (80–100)
Barthel Index at admission in IC units ^a^	20 (5–45)
Cognitive impairment (RLAS)^a^	7 (5–8)
Beginning rehabilitation in acute hospital	184 (47.9%)
Pressure ulcers ^a^	51 (13.3%)
Nasogastric feeding tube ^a^	48 (12.5%)
Percutaneous enteral gastrostomy ^a^	4 (1%)
Dysphagia ^a^	205 (53.4%)
Aphasia ^a^	187 (48.7%)
Length of stay at IC units (days) ^b^	61.6±45.6

Values are report as N (percentages), mean ± SD and median ± Interquartile range for categorical, quantitative and ordinal variables respectively.

^a^ Assessed at admission on Intermediate care.

^b^ Days of stay at Intermediate care.

Using cluster analysis, we defined 3 possible clusters or “rehabilitation complexity” profiles, which might be presented using the following paradigmatic phenotypes, according to baseline characteristics (Table 2).

**Table 2:** Clusters´ characteristics: variables included in the cluster analysis.

Characteristics	Post-stroke rehabilitation clusters		
	Lower Complexity with Caregiver (N = 169)	Moderate Complexity without Caregiver (N = 101)	Higher Complexity with Caregiver (N = 114)
Age	78.4 ± 7.8	76.2 ± 6.7	82.6 ± 7.9
Charlson Index	2.6 ± 2.2	3.0 ± 2.0	3.4 ± 2.3
Caregiver present^a^	Yes	No	Yes
Pre-stroke Barthel Index	92.0 ± 14.7	90.3 ± 15.4	75.6 ± 28.0
Stroke severity (NIHSS) ^a^	6.6 ± 4.8	8.2 ± 6.3	18.6 ± 7.7
Cognitive impairment (RLAS) ^a^^,^^b^	7.3 ± 1.1	6.6 ± 1.7	4.3 ± 1.5
Barthel Index at IC units admission	34.2 ± 22.9	36.5 ± 27.0	3.8 ± 7.3

Values are report as mean ± SD and mode for quantitative and categorical variables respectively.

^a^ Assessed at admission on Intermediate care.

^b^ Rancho Los Amigos Scale (RLAS), score from 1–8 points, describes coma—intact cognition.

1. Cluster 1, defined as “Lower Complexity with Caregiver”: patients under 80 years old, with good pre-stroke function, with a caregiver, low comorbidity, affected by a stroke of moderate severity, with a residual mild cognitive impairment and a high disability in ADLs at admission in IC;
2. Cluster 2, defined as “Moderate Complexity without Caregiver”: patients under 80 years old, with good pre-stroke function, without caregiver, moderate comorbidity, affected by a stroke of moderate severity, presenting a moderate cognitive impairment and a high dependence at admission in IC;
3. Cluster 3, defined as “Higher Complexity with Caregiver”: patients over 80 years old, with moderate pre-stroke disability in ADLs, with a caregiver, high comorbidity, who suffered a severe stroke, leading to a severe post-stroke cognitive and functional impairment.

**Fig 2:**
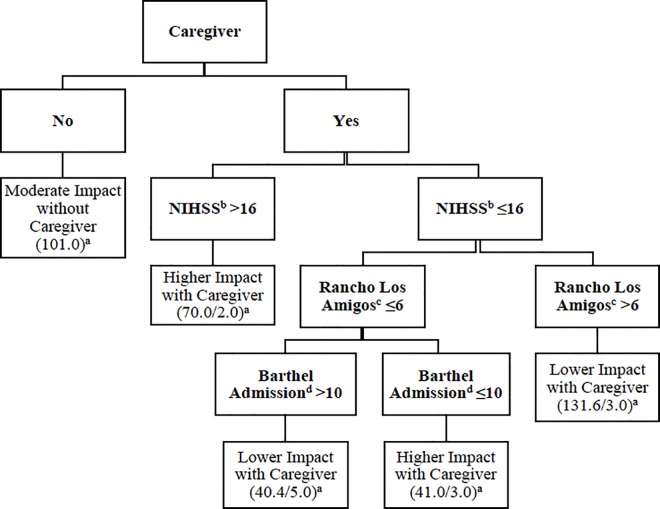
Decision tree for cluster´s allocation. ^a^ The total of patient classified into this cluster are represent by the first number; if there is a misclassification, a second number is shown, and if missing data exists, the algorithm assigned “half patient” to each cluster; ^b^ NIHSS: National Institute of Health Stroke Scale; a score of 16 define a severe stroke; ^c^ Rancho Los Amigos Scale measures cognitive function at admission (1–8, worse-better); at level 6, patient gives context appropriate, goal-directed responses, present recent memory problems; ^d^ Barthel index of 10 or less indicates severe disability.

In order to offer a practical tool to better describe our profiles and to potentially use the cluster allocation in the clinical practice, we designed a decision tree, based on the variables included in the cluster analysis (Fig 2). A very high agreement between the allocation obtained using the decision tree and the cluster analysis was observed (96.6%).

We compared the three groups for baseline characteristics not included in the cluster analysis (Table 3): the “Lower complexity” group was the group in which a higher percentage of patients began rehabilitation at acute hospitals; the “Moderate Complexity” group had more lifestyle risk factors and a lower percentage of patients in this cluster had begun rehabilitation treatment in the acute care hospital; finally, the “Higher Complexity” group, tended to include older participants with higher pre-stroke disability, had more women, less prevalence of lifestyle risk factors (smoking and alcohol consumption), and a higher rate of previous institutionalization. Patients of this group experienced more clinical complications related to stroke (pressure ulcers, dysphagia, and aphasia) and had higher functional impairment at discharge.

**Table 3:** Clusters´ characteristics: variables not included in the cluster analysis.

Characteristics	Post-stroke rehabilitation clusters			
	Lower Complexity with Caregiver (N = 169)	Moderate Complexity without Caregiver (N = 101)	Higher Complexity with Caregiver (N = 114)	p on trend
Female	82 (48.5%)	45 (44.6%)	68 (59.6%)	0.64
Smoke consumption	68 (40.2%)	51 (50.5%)	29 (25.4%)	<0.001
Alcohol consumption	18 (10.7%)	22 (21.8%)	12 (0.5%)	0.03
Dementia	28 (16.7%)	18 (17.8%)	33 (28.9%)	0.12
Cerebrovascular disease	51 (30.2%)	30 (29.7%)	48 (42.1%8)	0.72
Diabetes Mellitus	63 (37.3%)	39 (38.6%)	39 (34.2%)	0.78
Dyslipidemia	80 (47.3%)	48 (47.5%)	41 (36.0%)	0.12
Previous Institutionalization	4 (2.4%)	3 (3.0%)	4 (3.5%)	0.93
Ischemic Stroke	136 (80.5%)	81 (80.2%)	94 (82.5%)	0.82
Beginning rehabilitation at acute hospital	103 (61.7%)	45 (48.9%)	76 (32.1%)	<0.001
Pressure Ulcers ^a^	14 (8.3%)	10 (9.9%)	27 (23.7%)	0.001
Nasogastric feeding tube ^a^	6 (3.6%)	9 (8.9%)	33 (28.9%)	<0.001
Percutaneous enteral gastrostomy ^a^	2 (1.2%)	0 (0.0%)	2 (1.8%)	0.24
Dysphagia ^a^	69 (41.3%)	49 (48.5%)	87 (77.7%)	<0.001
Aphasia^a^	64 (37.9%)	45 (44.6%)	78 (68.4%)	<0.001
Barthel Index at discharge from IC units	60 (42.5–85)	60 (35–86.2)	10 (5–35)	<0.001

Values are report as N (percentage) and median (Interquartile range, IQR) for categorical and ordinal variables respectively, p <0.05 was consider statistical significant.

^a^ Assessed at admission on Intermediate care units.

Using ANCOVA (Table 4), the “Lower Complexity” group showed the greatest mean functional improvement and relative functional gain. Mean differences in functional outcomes between the “Lower Complexity” and the “Higher Complexity” group were statistically significant. No differences were shown regarding LOS across groups. Mean rehabilitation efficiency was higher in “Lower Complexity” patients, and lowest in “Higher Complexity” patients, who also had a higher proportion of new institutionalizations.

**Table 4:** Association between the clusters and outcomes.

Outcomes	Post-stroke rehabilitation clusters			p-value	p-value
	Lower Complexity with Caregiver (N = 169)	Moderate Complexity without Caregiver (N = 101)	Higher Complexity with Caregiver (N = 114)		
	ANCOVA (adjusted by sex)				LINEAR REGRESSION
Functional improvement	21.6±29.0	18.2±25.4	8.6±18.6	<0.001^a^^,^^b^	0.007^a^^,^^b^
Relative functional gain	0.4±0.6	0.40±0.8	0.2±0.4	0.033^a^	0.156^a^^,^^b^
Length of stay	58.02±43.1	68.7±40.8	60.5±52.6	0.189	0.361^c^
Rehabilitation efficiency	0.47±1.3	0.4±0.8	0.1±0.6	0.005^a^	0.064^a^^,^^b^
	CHI-SQUARE (linear trend)				LOGISTIC REGRESSION
New Institutionalization	17.8 (28)	27.2 (25)	34.6 (36)	0.008^a^	0.144

Values are report as mean±SD or percentages (N) for continuous or dichotomous outcomes, respectively. Functional improvement was calculated as BI at discharge minus BI at admission; Relative functional gain was calculated as Functional improvement divided by (pre-stroke BI minus BI at admission); Rehabilitation efficiency was calculated as Functional improvement divided by Length of stay. ANCOVA models were adjusted by sex; multivariable regression models (linear regression for all the outcomes but logistic regression for new institutionalization) were adjusted by sex, type of stroke, dysphagia, beginning of rehabilitation in the acute hospital. Differences according to post-hoc Bonferroni analysis after ANCOVA model, and contrasts between clusters in logistic regression models showed:

^a^ Difference between “Lower Complexity with Caregiver” and “Higher Complexity with Caregiver”, p <0.05.

^b^ Difference between “Moderate Complexity without Caregiver” and “Higher Complexity with Caregiver”, p <0.05.

^c^ Difference between “Lower Complexity with Caregiver” and “Moderate Complexity without Caregiver”, p <0.005.

The post-hoc analysis of Bonferroni showed a statistically significant difference between the “Lower Complexity” cluster and the “Higher Complexity” cluster on functional improvement (mean difference = 12.35, 95%CI = 4.96–19.73, p<0.001), relative functional gain (mean difference = 0.24, 95%CI = 0.02–0.46, p = 0.027), rehabilitation efficiency (mean difference = 0.4, 95%CI = 0.1–0.69, p = 0.004) and new institutionalization (17.8% on the first group and 34.6% on the second one, p = 0.005). Comparing the “Moderate Complexity” and the “Higher Complexity” groups, there were differences in functional improvement (mean difference = 8.7, 95%CI = 0.34–17.02), p = 0.038). We found no significant differences in LOS between the different clusters. Comparing the “Lower Complexity” and “Moderate Complexity” clusters, no significant differences in the outcomes were shown, but there was a trend towards higher LOS and institutionalizations in the “Moderate Complexity” group.

In multivariable linear regression models, a progressively higher complexity of the clusters showed a statistically significant association with functional improvement, but not with other outcomes (Table 4). We repeated logistic regression models using dichotomized outcomes, and these generally confirmed the results of the linear regression models: comparing the “Lower” and the “Higher Complexity” clusters, we found that the first group had a fivefold increased chance of improving ≥20 points in the BI (OR = 4.68, 95%CI = 2.54–8.63, p<0.001), a fourfold increased chance to recover more than 60% of lost functional capacity due to stroke (OR = 4.42, 95%CI = 1.81–10.75, p = 0.001) and an eightfold chance to have a greater rehabilitation efficiency (OR = 7.88, 95%CI = 3.65–17.03, p<0.001). After comparing the “Moderate Complexity” and the “Higher Complexity” clusters, the first had a threefold increased chance of improving ≥20 points in the BI (OR = 3.0 95%CI = 1.52–5.87), p = 0.001), to recover more than 60% of lost functional capacity (OR = 3.45, 95%CI = 1.31–9.04, p = 0.012), a fourfold chance to have a better rehabilitation efficiency (OR = 3.87, 95%CI = 1.69–8.89, p = 0.001) and also less chance of shorter LOS (OR = 0.37, 95%CI = 0.19–0.73, p = 0.004). Finally, after comparing “Lower complexity” and “Moderate complexity” clusters, the second one had a lower chance of shorter LOS (OR = 0.41, 95%CI = 0.23–0.75), no other differences between this two clusters were found. No difference on pair-wise comparison was found for new institutionalization and LOS.

## Discussion

In our sample of older stroke survivors, we used cluster analysis to identify three possible stroke rehabilitation profiles, and, based on the obtained results, we built a decision tree for easier practical classification. The three groups differed in baseline characteristics, functional status and outcomes. Stroke severity at IC admission, post-stroke disability, cognitive impairment and presence of a caregiver seemed the main characteristics in order to assign patients to the clusters. In multivariable models adjusted for different potential confounders, an association between cluster assignment and functional recovery was observed. In pair-wise comparisons, the less complex profiles, compared to the more complex one, also showed a greater relative functional gain and more rehabilitation efficiency.

The baseline characteristics used to build the clusters are recognized as strong predictors of stroke outcomes [29,30]. The importance of functional impairment (pre and post stroke disability) in defining clusters and initial stroke severity is consistent with previous studies [23,24]. However, there is no consensus on when measuring functional status, with a recent study describing a good correlation between its assessment during the first five days and the independence for activities of daily living at six months [31]. The average latency time between stroke diagnosis and admission to IC unit in Catalonia is one week, which reinforces the importance of functional assessment at IC admission [31,32]. On the other hand, some authors report that the NIHSS evaluation between days 2 and 9 remains stable, so that this timeframe is in line with our measurement, and it’s an independent predictor of 6 months. Our work also highlights the importance of caregivers’ presence [26]: patients without a caregiver tend to stay longer in IC and, regardless of the severity of stroke and pre and post functional dependency, the lack of a caregiver directly orients to moderate complexity.

Other international studies, from China and the Netherlands, used cluster analysis to describe profiles of stroke survivors. Despite socio-cultural healthcare systems differences, there are similarities with our results [33,34]. In all three studies, disability at admission emerges as a key characteristic to identify clusters. Similar to ours, the other two studies also identified one specific cluster with a more severe impact of stroke and functional consequences (defined “Higher Complexity with Caregiver” in our work, and “Poor condition” in the others studies). However, this cluster showed different trajectories of recovery in the two studies. In the study by Buijck et al, the “Poor condition” cluster had a relative greater functional improvement, possibly explained by a floor effect (higher chance of improving); conversely, compared with our other clusters, we found a worse improvement in the “Lower Complexity with Caregiver” group, possibly due to the previous functional impairment and more severe stroke [33]. Differences in healthcare and rehabilitation resources might also contribute to these different results.

Important outcomes differed between the clusters, suggesting the validity of the clustering. We found differences between groups (linear or in pair-wise comparisons) for all the main functional-related outcomes, including relative gain, and efficiency. LOS has been criticized as an outcome measure in stroke trials as it is biased by early mortality, institutionalization and social variables; in these sense, in our sample, it seems that the absence of caregiver could contribute to have longer LOS. If these results are proved to be true, it could have implications on social and healthcare resources allocation. Regarding institutionalization, which represent a negative post-stroke outcome, we did not find a clear association, but a trend towards a higher occurrence in more complex patients was found. It is also possible that institutionalization occur later for some patients, after a first attempt to care for the person at home is made.

We acknowledge limitations in our study. We did not collect information about stroke acute treatments (fibrinolysis or revascularization) and complications, nor about specific stroke classifications besides ischemic/hemorrhagic. Regarding functional assessment, other scales, such as the modified Rankin Scale, could be used instead of BI, but there is no consensus on a “gold standard”. Finally, RLAS is not commonly used in Spanish IC units. Strengths of our study include the multicenter design with a large, “real world” population and the comprehensive assessment. The combined statistical methods, with the proposition of a practical approach to identify patients’ profile, can be also considered as innovative.

## Conclusion

In our relatively large multi-centric sample of older stroke survivors admitted to post-acute geriatric rehabilitation, a common comprehensive assessment, that could be applied easily at admission at any stroke rehabilitation unit, was the basis for the identification of three complexity rehabilitation profiles, which showed differences in functional recovery. In the context of limited healthcare resource and potentially increasing demand of stroke services, understanding profiles of older adults admitted to IC after a stroke may help clinical and policy decision making. We speculate that, if our results will be confirmed by other studies, the early identification of different clusters, based on a standard assessment and supported by a visual algorithm to assign a newly admitted patient to a specific IC rehabilitation profile, could be used to test and eventually offer intervention programs tailored for patients’ needs and expected outcomes. Among other potential uses, the identification of profiles might help to estimate and inform patients and caregivers about prognosis and goal setting, and, if complemented by further economic analyses, to improve planning, policy and resource allocation.

## Supporting Information

S1 Data Post-stroke older adults admitted to intermediate care units in Catalonia during 2010. (XLS)

## Abbreviations

ADL: Activities of daily living
BI: Barthel Index
HCC: Higher Complexity with Caregiver
IC: Intermediate Care
IRP: Intensive Rehabilitation Program
LCC: Lower Complexity with Caregiver
LOS: Length of Stay
MCN: Moderate Complexity without Caregiver
NIHSS: National Institute of Health Stroke Scale
RLAS: Rancho Los Amigos Scale
